# Supplementary material for: Investigation of Laser-Induced Graphene (LIG) on a Flexible Substrate and Its Functionalization by Metal Doping for Gas-Sensing Applications
Source: Int J Mol Sci. 2024 Jan 18;25(2):1172. doi: 10.3390/ijms25021172 (PMC10816167; doi:10.3390/ijms25021172)
Supplement: Supplementary file 1 [file ijms-25-01172-s001.zip › ijms-2824028-supplementary.pdf]

Supporting Information for

Investigation of Laser-Induced Graphene (LIG) on a Flexible Substrate and Its Functionalization by Metal Doping for Gas-Sensing Applications

Dongwook Kwak [1], Hyojin Kim [1], Seunghun Jang [2], Byoung Gak Kim [1], Donghwi Cho [1], Hyunju Chang [2] and Jeong-O Lee [1,*]

[1] Advanced Materials Division, Korea Research Institute of Chemical Technology, 141 Gajeongro, Yuseong-gu, Daejeon 34114, Republic of Korea; dkwak@krict.re.kr (D.K.); hjkim26@krict.re.kr (H.K.); bgkim@krict.re.kr (B.G.K.); roy.cho@krict.re.kr (D.C.)

[2] Data Research Center, Korea Research Institute of Chemical Technology, 141 Gajeongro, Yuseong-gu, Daejeon 34114, Republic of Korea; jang@krict.re.kr (S.J.); hjchang@krict.re.kr (H.C.)

* Correspondence: jolee@krict.re.kr; Tel.: +82-42-860-7336; Fax: +82-42-860-7508

List of Figures

Figure S1. Comparison of the responses of the P-LIG device to 1000 ppm NO_2 while varying temperature from 50°C to 150°C.

Figure S2. (a,b) Cross-sectional SEM images of the P-LIG, (c,d) cross-sectional SEM and EDS images of the 4-nm-thick Au-doped LIG, and (e-g) EDS mapping images of the 2-nm-, 4-nm-, and 8-nm-thick Au-doped LIGs.

Figure S3. High resolution XPS spectra of Ag, Al, Au, Cu, In, and Pd metal dopants.

Figure S4. Electrical resistance variance of the P-LIG device at temperatures from RT to 150°C.

Figure S5. Comparison plot of gas responses of the P-LIG device to different concentrations of NO_2 and NH_3.

Figure S6. Gas responses of the P-LIG device to different concentrations of (a) NO_2 and (b) NH_3.

Figure S7. Gas response plots of the P- and M-LIG devices to 1000 ppm of NO_2 over three on/off cycles.

Figure S8. Gas response plots of the P- and M-LIG devices to 1000 ppm of NH_3 over three on/off cycles.

Table S1. XPS quantification results for the P- and M-LIG devices.

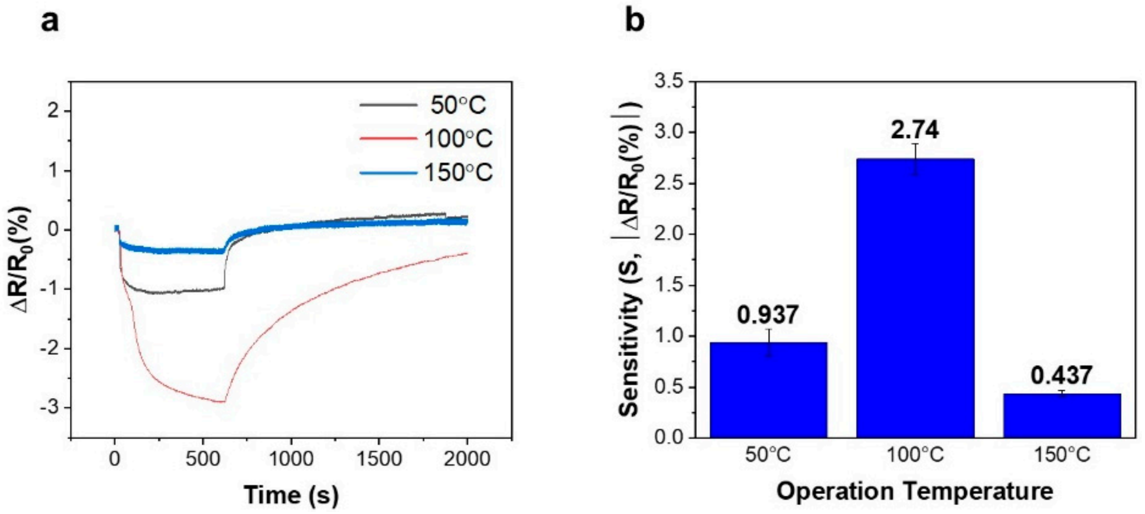

Figure S1. Comparison of the responses of the P-LIG device to 1000 ppm NO_2 while varying temperature from 50℃ to 150℃.

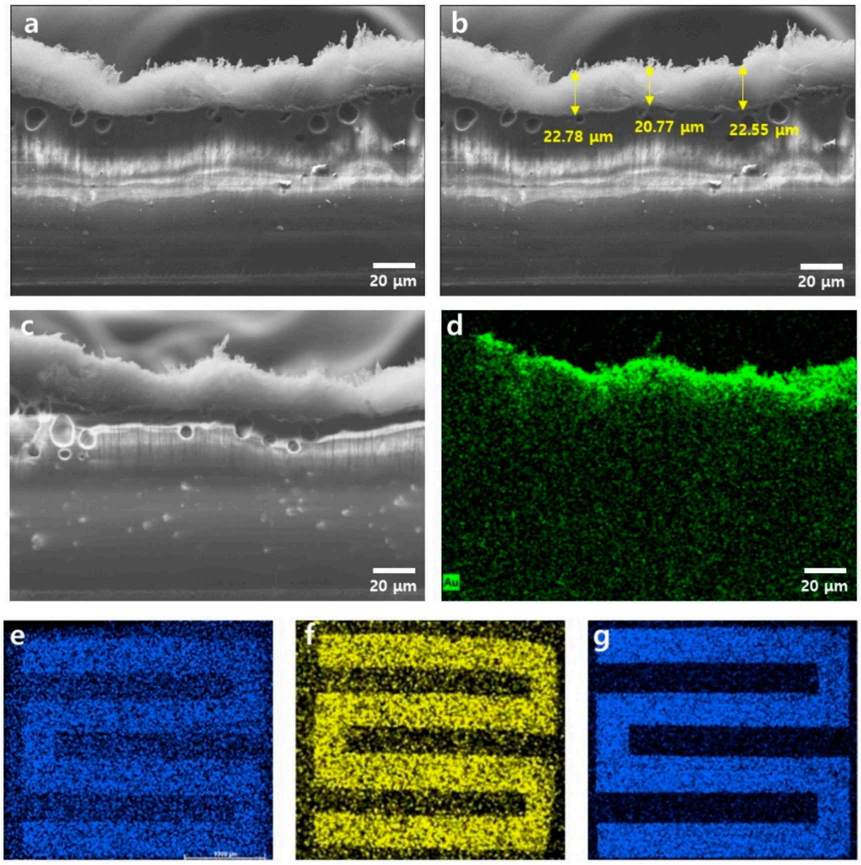

Figure S2. (a,b) Cross-sectional SEM images of the P-LIG, (c,d) cross-sectional SEM and EDS images of the 4-nm-thick Au-doped LIG, and (e-g) EDS mapping images of the 2-nm-, 4-nm-, and 8-nm-thick Au-doped LIGs.

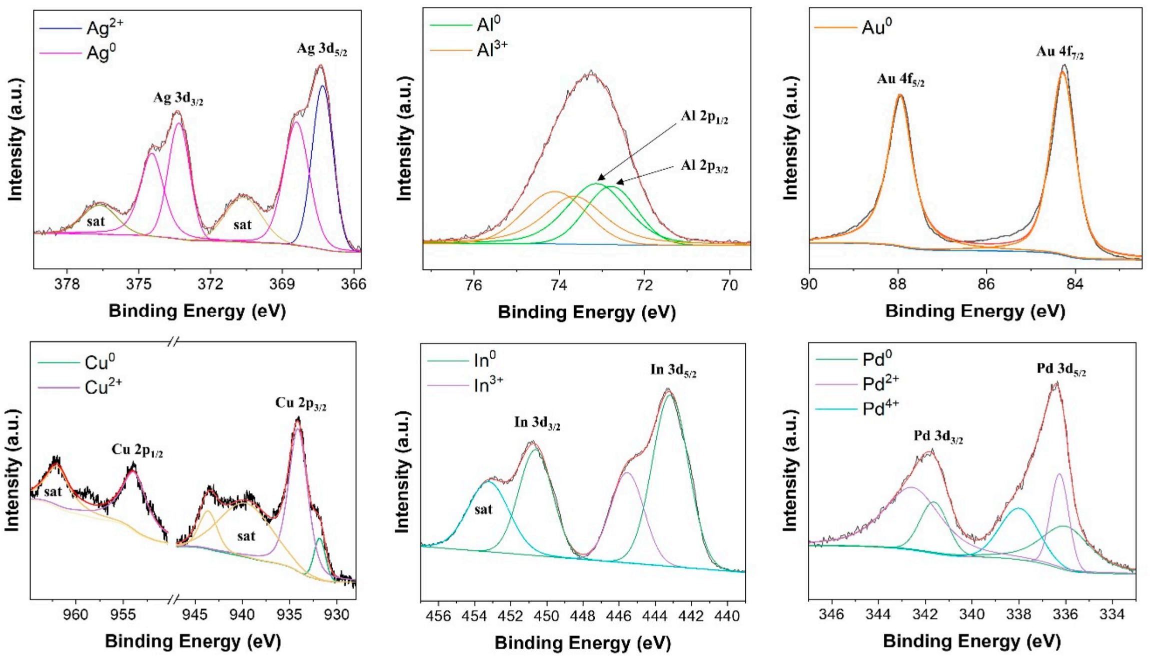

Figure S3. High resolution XPS spectra of Ag, Al, Au, Cu, In, and Pd metal dopants.

Table S1. XPS quantification results for the P- and M-LIG devices.

Sample	XPS element concentration (%)				XPS atomic concentration (%)			
	C	O	N	metal	C	O	N	metal
pristine	94.9	5.1	-	-	96.1	3.9	-	-
Ag	66.4	17.0	2.3	10.2	79.2	15.3	2.4	1.4
Au	56.3	16.4	2.6	21.6	77.1	16.9	3.1	1.8
Al	50.7	31.4	1.8	14.5	61.1	28.4	1.9	7.8
Cu	58.2	22.5	1.7	15	72.5	21.1	1.8	3.5
In	53.1	17.2	1.6	26.9	75.3	18.3	1.6	4.0
Pd	52.3	26.1	4.1	8.3	65.4	24.5	4.4	1.2

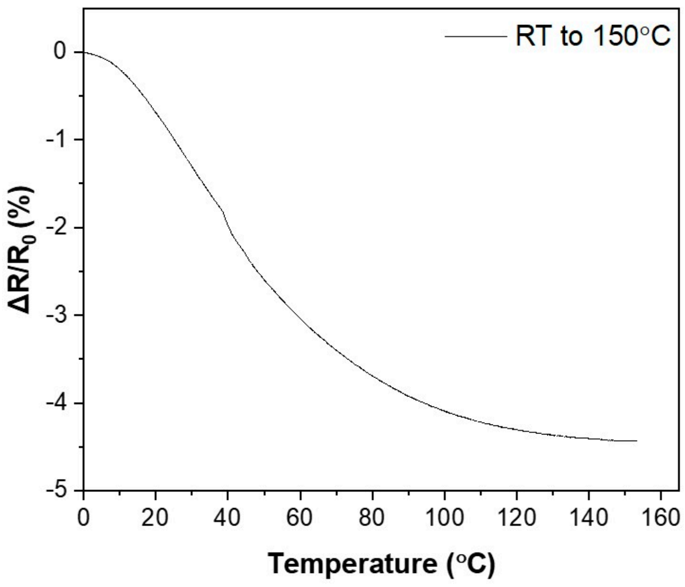

Figure S4. Electrical resistance variance of the P-LIG device at temperatures from RT to 150°C.

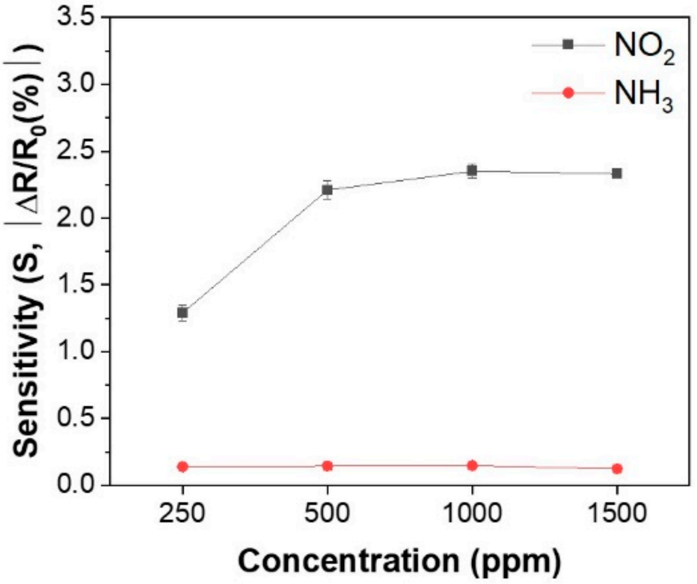

Figure S5. Comparison plot of gas responses of the P-LIG device to different concentrations of NO_2 and NH_3.

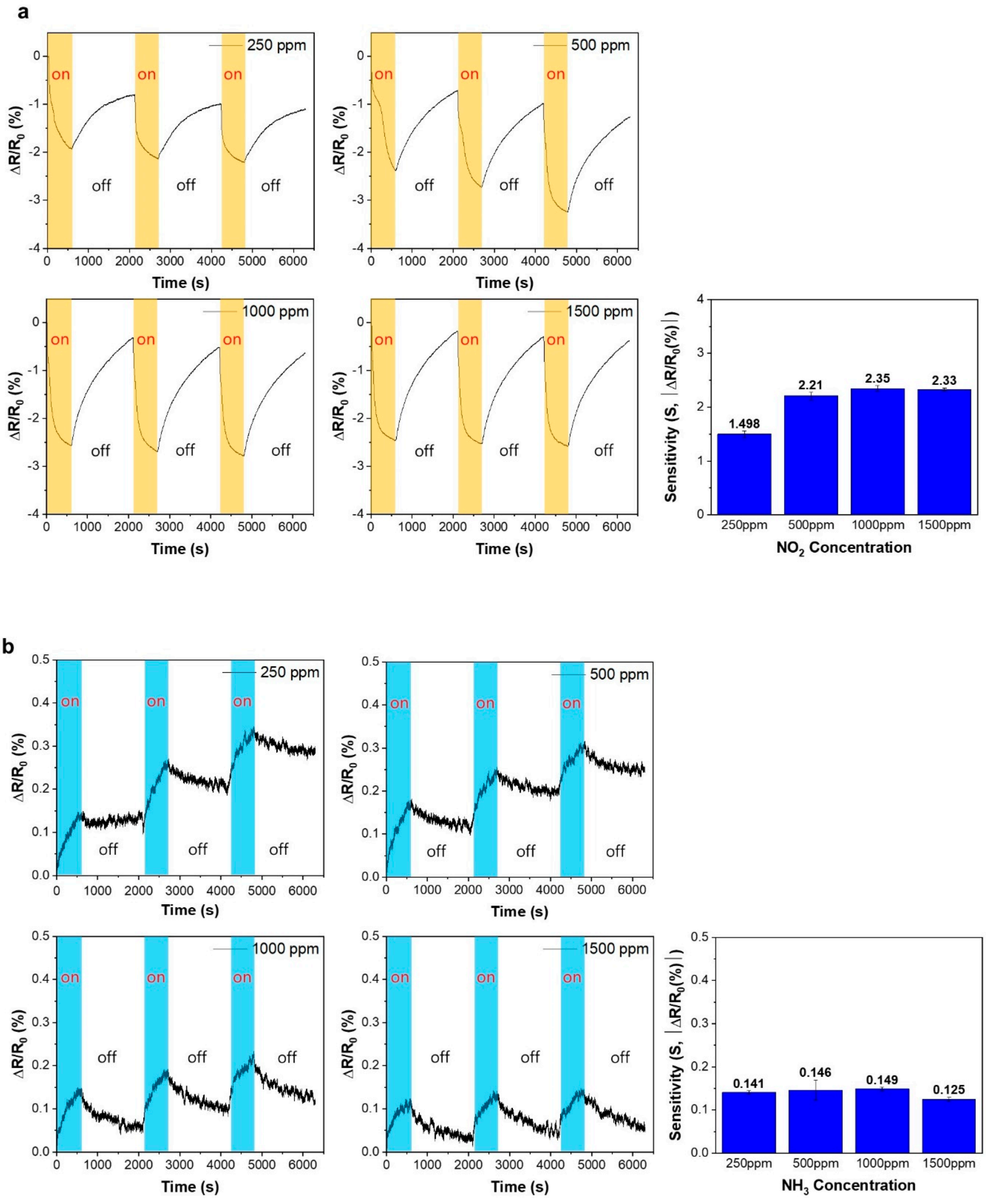

Figure S6. Gas responses of the P-LIG device to different concentrations of (a) NO_2 and (b) NH_3.

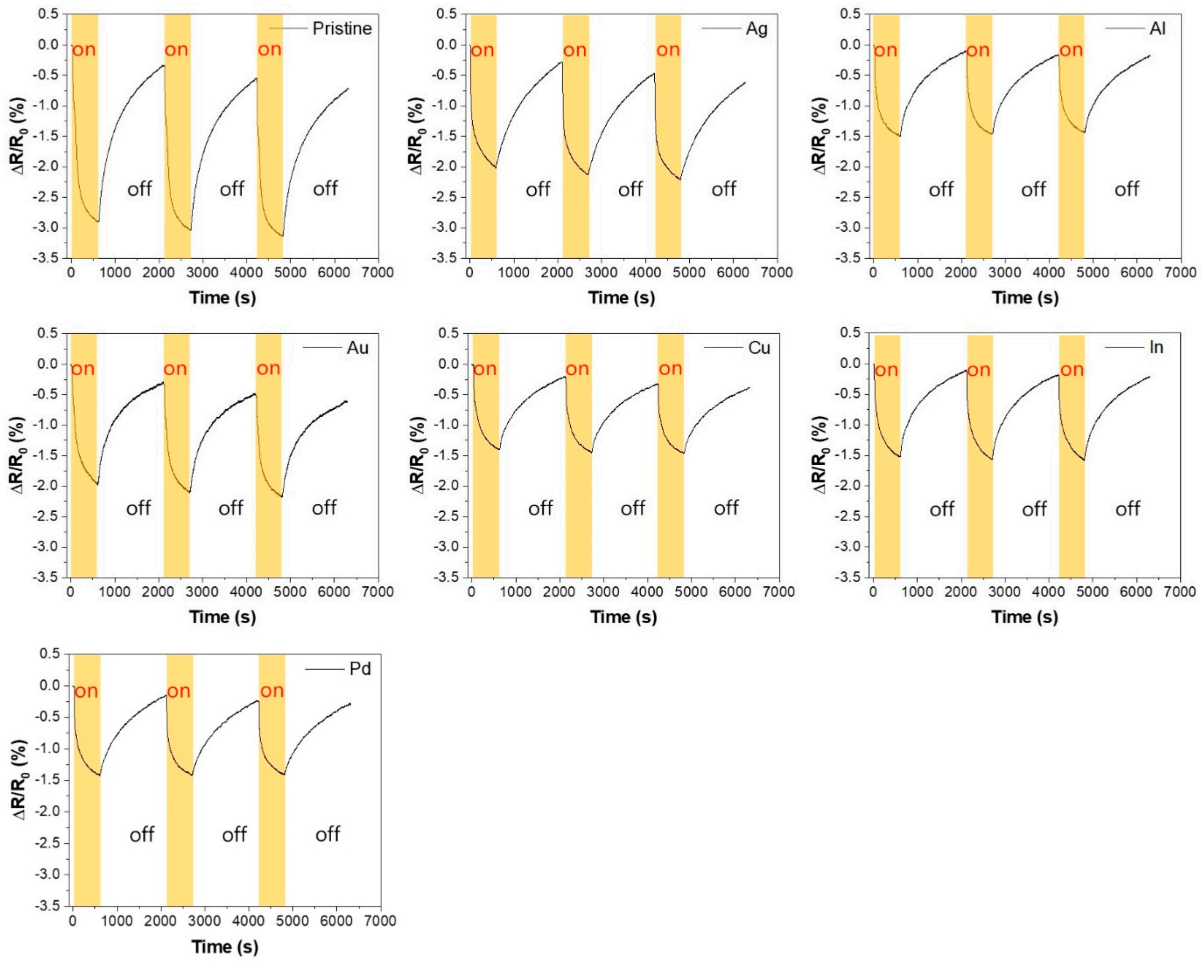

Figure S7. Gas response plots of the P- and M-LIG devices to 1000 ppm of NO_2 over three on/off cycles.

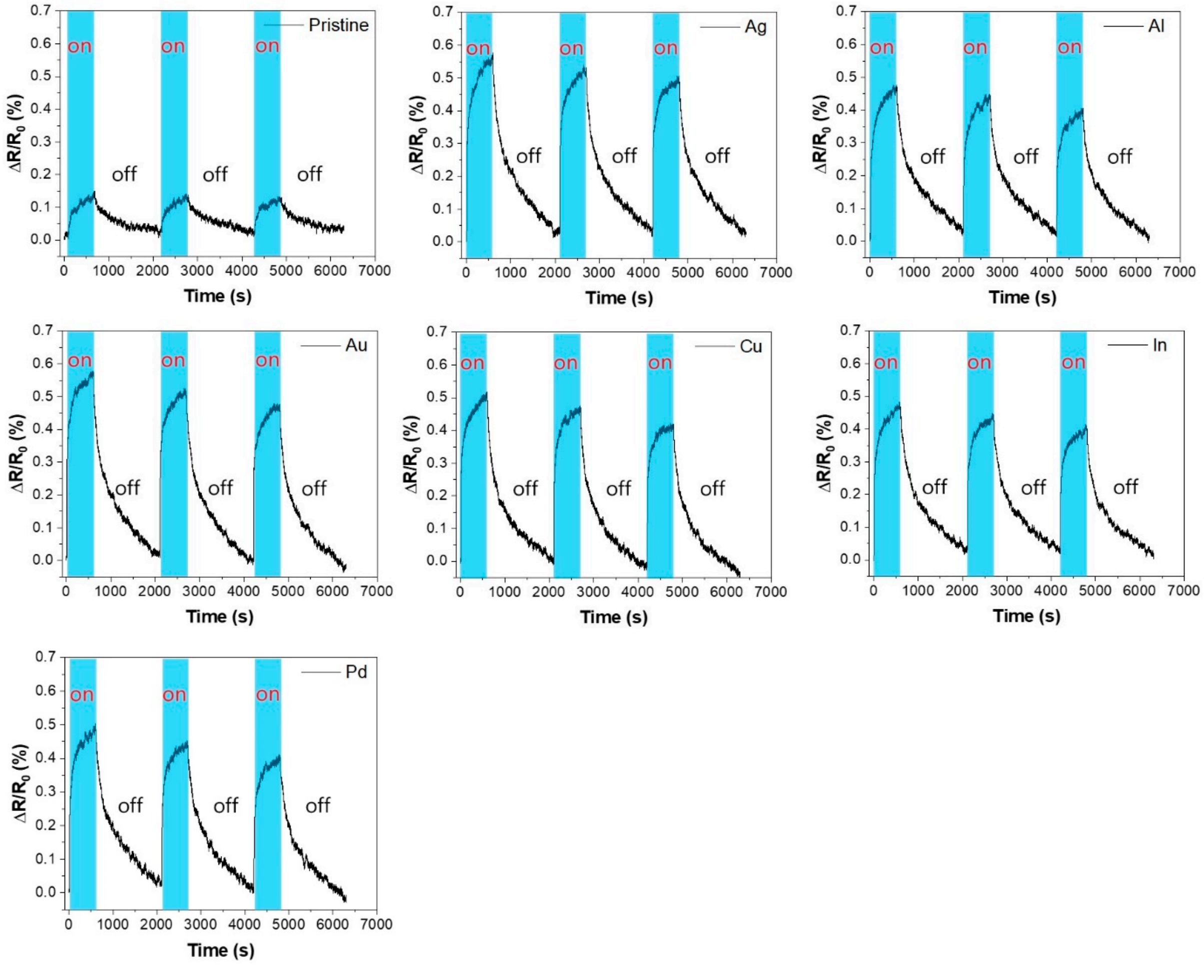

Figure S8. Gas response plots of the P- and M-LIG devices to 1000 ppm of NH_3 over three on/off cycles.
